# Clustering multilayer omics data using MuNCut

**DOI:** 10.1186/s12864-018-4580-6

**Published:** 2018-03-14

**Authors:** Sebastian J. Teran Hidalgo, Shuangge Ma

**Affiliations:** 1 Department of Biostatistics, Yale School of Public Health, 60 College Street, New Haven, 06520 USA; 2 Department of Statistics, Taiyuan University of Technology, 79 Yingze W St, Wanbailin Qu, Taiyuan Shi, Shanxi Sheng, 030024 People’s Republic of China

**Keywords:** Multilayer omics data, Clustering, NCut

## Abstract

**Background:**

Omics profiling is now a routine component of biomedical studies. In the analysis of omics data, clustering is an essential step and serves multiple purposes including for example revealing the unknown functionalities of omics units, assisting dimension reduction in outcome model building, and others. In the most recent omics studies, a prominent trend is to conduct multilayer profiling, which collects multiple types of genetic, genomic, epigenetic and other measurements on the same subjects. In the literature, clustering methods tailored to multilayer omics data are still limited. Directly applying the existing clustering methods to multilayer omics data and clustering each layer first and then combing across layers are both “suboptimal” in that they do not accommodate the interconnections within layers and across layers in an informative way.

**Methods:**

In this study, we develop the MuNCut (Multilayer NCut) clustering approach. It is tailored to multilayer omics data and sufficiently accounts for both across- and within-layer connections. It is based on the novel NCut technique and also takes advantages of regularized sparse estimation. It has an intuitive formulation and is computationally very feasible. To facilitate implementation, we develop the function muncut in the R package NcutYX.

**Results:**

Under a wide spectrum of simulation settings, it outperforms competitors. The analysis of TCGA (The Cancer Genome Atlas) data on breast cancer and cervical cancer shows that MuNCut generates biologically meaningful results which differ from those using the alternatives.

**Conclusions:**

We propose a more effective clustering analysis of multiple omics data. It provides a new venue for jointly analyzing genetic, genomic, epigenetic and other measurements.

**Electronic supplementary material:**

The online version of this article (10.1186/s12864-018-4580-6) contains supplementary material, which is available to authorized users.

## Background

In biomedical studies, omics profiling is now routinely conducted. In the analysis of omics data, clustering is an essential step. Clustering results can be used in multiple ways. For example, they can suggest the unknown functionalities of omics units, with those in the same clusters likely to have related biological functions [1]. Clustering can also assist dimension reduction/variable selection in outcome model building [2]. A large number of clustering methods have been developed under both “classic” low-dimensional settings and high-dimensional settings for omics data [3, 4]. The existing literature is too vast to be reviewed here. For relevant discussions, we refer to [5–7] and others.

Complex biological processes involve changes at the genetic, epigenetic, genomic, and other levels. Most recently, a prominent trend in biomedical research is to conduct multilayer profiling, which collects multiple types of omics measurements on the same subjects. A representative example is TCGA (The Cancer Genome Atlas), which is organized by the NIH/NCI and has data publicly available. In TCGA, for multiple cancer types, data have been collected on mRNA gene expression, DNA methylation, microRNA, copy number variation, protein expression, and others. Such multilayer data have been analyzed in recent studies. For example, in [8] and others, they lead to disease outcome models with better predictive power than analyzing a single layer of data. In [9] and others, more reliable omics markers missed by single-layer studies are identified. However, our literature search suggests that there is still insufficient attention to clustering analysis with multilayer omics data.

Clustering analysis with multilayer omics data is challenging. Directly applying the existing clustering methods may not be appropriate. The interconnections within layers (for example, among gene expressions) and those across layers (for example, between gene expressions and CNVs) are different both biologically and statistically. The existing clustering methods are mostly designed for “homogeneous” variables and cannot sufficiently accommodate such differences. Another possible strategy is to first cluster within each layer and then combine clusters across layers. This strategy ignores the regulations across layers and does not use all available information. The ineffectiveness of such strategies can be partly seen in our numerical study.

The goal of this study is to fill the knowledge gap by developing a clustering method tailored to multilayer omics data. Considering the fast increasing popularity of multilayer omics data, essential role of clustering analysis, and lack of multilayer omics data clustering methods, the proposed study is warranted. It advances from the existing literature in multiple ways. Compared to the existing clustering analyses with a single layer of omics data, the data structure is much more complicated: there are multiple types of variables, and the connections among variables are different. This study also differs from the existing ones on multilayer omics data. Some published studies use a multilayer representation of omics data to find meaningful subgroups of subjects [10–13]. Representative examples include the iCluster [2] and Similarity Network Fusion (SNF) [14], both of which cluster patients into different subgroups. In another recent study [15], data on patients, genes, and drugs are jointly analyzed for clustering. The output of this study is three different subgroups of the three different data types. There are also multilayer studies that focus on marker identification and model building. For example, multiple layers of protein-genetic interactions have been aggregated to form a smaller set of layers [16]. There are also recent works on graph measures of centrality developed specifically for multilayer data, including for example measures of node centrality [17] and methods for community detection [18]. Data used in some of the aforementioned studies are similar to the present study. However, the analysis goals are quite different. Specifically, in the aforementioned clustering analyses, the goal is to cluster subjects (patients), whereas in the present study, the goal is to cluster omics measurements. With this difference, the existing clustering methods are not applicable to the present problem. In our numerical studies, we have attempted to employ these methods but obtained failing results (details available from the authors and omitted here).

This study has a different goal from the aforementioned studies and targets at clustering multiple types of omics measurements. Methodological development in this study is challenging and tailored to the special characteristics of multilayer data. The proposed method is built on the NCut technique [19], which has multiple advantages over some of the existing techniques (for example, by making fewer and weaker data/model assumptions) but has not been extensively applied in omics studies, and significantly advances from it. As such, this study may also have independent methodological value. Numerical study will show that the proposed method is computationally much feasible and outperforms multiple relevant competitors. Overall, this study can provide a useful new venue for a practically important problem.

## Methods

**Fig. 1:**
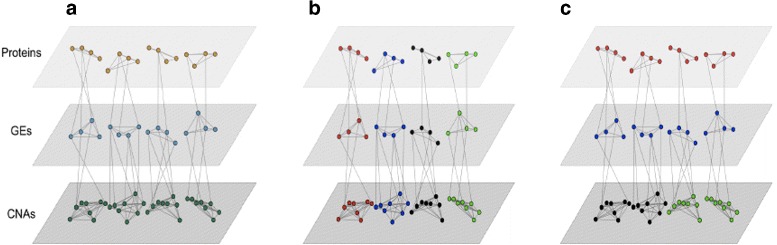
Multilayer omics data and clustering. Three data types are considered: proteins in the upper layer; gene expressions in the middle layer; and CNVs in the lower layer. One dot represents one variable. Two dots are connected by a line if the corresponding variables are interconnected). Left panel: the true data structure with four clusters. Middle panel: MuNCut clustering. Right panel: K-means clustering. For K-means and MuNCut, different clusters are represented using different colors. **a** Truth. **b** MuNCut. **c** K-means

As a representative example, consider a profiling study that collects measurements on copy number variations (CNVs), gene expressions (GEs), and proteins. Data with other types of measurements can be analyzed in the same way. The schematic plot of the data structure is shown in Fig. 1. The three types of measurements make three layers. The bottom layer consists of CNVs, the middle layer consists of GEs, and the upper layer consists of proteins. The regulatory relationship between different types of omics measurements has been studied long [20–22]. Simply put, as shown in Fig. 1, there are multiple “channels”, which correspond to different biological functionalities. Within each channel, a small number of CNVs in the lower layer regulate a small number of GEs in the middle layer, which encode a small number of proteins in the upper layer. In clustering analysis with a single type (layer) of measurements, say for example GEs, the goal is to put interconnected GEs in the same cluster. With multiple types (layers) of measurements, there are two types of interconnections: within layers and across layers. In clustering analysis with multilayer data, our first goal is still to put interconnected CNVs (GEs, proteins) in the same cluster. Unique to multilayer data, our second goal is to put tightly interconnected measurements in different layers also in the same cluster. As such, as shown in Fig. 1, one resulting cluster corresponds to one channel and consists of multiple types of omics measurements. When limited to a single-layer, the obtained clustering structure is comparable to that of the existing methods. As to be shown below, the proposed method includes the existing one as a special case and can be more informative by considering multilayers.

### MuNCut

Denote *Z*=(*Z*_1_,…,*Z*_*q*_),*Y*=(*Y*_1_,…,*Y*_*p*_), and *X*=(*X*_1_,…,*X*_*r*_) as the length *q*, *p*, and *r* vectors of proteins, GEs, and CNVs, respectively. Assume that data have been properly processed. With multilayer data, as described above, both within- and across-layer connections need to be considered.

**NCut clustering within the same layers** First consider CNVs. Denote ***W***_*C*_=(*w*_*jl,c*_)_*r*×*r*_ as the weight matrix, where the non-negative element *w*_*jl,c*_ measures the similarity between CNVs *j* and *l*. Following published studies [19, 23], we set *w*_*jl,c*_ equal to the Gaussian kernel. In the literature, multiple similarity measures have been proposed. We adopt this specific definition because of its simplicity and effectiveness demonstrated in published studies. Denote *A*_1,*C*_,…,*A*_*K,C*_ as a partition of {1,…,*r*} which leads to *K* disjoint CNV clusters. Here in the subscript, “*C*” is used to represent CNV. For *A*_*k,C*_, denote $A_{k,C}^{c}$ as its complement set. Consider the NCut measure

1 $$\begin{array}{@{}rcl@{}} {}\text{NCut}_{C}= \sum \limits_{k=1}^{K}\frac{\text{cut}\left(A_{k,C},A_{k,C}^{c};\boldsymbol{W}_{C}\right)} {\text{cutvol}(A_{k,C}; \boldsymbol{W}_{C})}, \end{array} $$

where

2 $$ \text{cut}\left(A_{k,C},A_{k,C}^{c};\boldsymbol{W}_{C}\right)=\sum \limits_{j\in A_{k,C},l \in A_{k,C}^{c}} w_{jl,c},  $$

and

3 $$ \text{cutvol}(A_{k,C}; \boldsymbol{W}_{C})=\sum \limits_{j,l \in A_{k,C}} {w}_{jl,c}.  $$

In a similar way, we can define the NCut measures for GEs and proteins and denote them as NCut_*G*_ and NCut_*P*_, respectively. Note that each layer has its own weight matrix, namely ***W***_*C*_,***W***_*G*_, and ***W***_*P*_. Overall, define the single-layer NCut measure as

4 $$\begin{array}{@{}rcl@{}} \text{NCut}_{single}=\text{NCut}_{C}+\text{NCut}_{G}+\text{NCut}_{P}. \end{array} $$

The optimal cutting is defined as the one that minimizes NCut_*single*_. Note that NCut_*single*_ does not take into account the regulations (interconnections) across layers, and working with this measure is equivalent to conducting the NCut clustering with each layer individually.

Remarks The NCut technique is originally developed in imaging and other scientific fields [24, 25] and more recently applied to genetic and other data types [26]. It may have multiple advantages over the alternatives. For example, the cutting step is relatively independent of the similarity/distance construction. Without making restrictive assumptions on the similarity measure and underlying data distributions and models, it enjoys broad applicability. Both the numerator and denominator in (1) have lucid interpretations, with the numerator measuring the across-cluster similarity and the denominator measuring the within-cluster similarity. As such, NCut is built on a sound statistical principle: it minimizes the across-cluster similarity while maximizing the within-cluster similarity. In addition, data analysis in this and other studies suggests that it is computational affordable, even with high data dimensionality.

**NCut clustering across layers** In the above subsection, we have focused on the interconnections (similarity) for omics measurements within the same layers. Now we consider the interconnections between omics measurements belonging to different layers (for example, CNVs and GEs). Following the literature [27], we first adopt a regression-based approach to describe the regulations. Specifically, consider the models:

5 $$\begin{array}{@{}rcl@{}} Y=X\boldsymbol{\beta_{1}}+\epsilon_{1}, ~~ Z=Y\boldsymbol{\beta_{2}}+\epsilon_{2}, \end{array} $$

where ***β***_***1***_ and ***β***_***2***_ are the *r*×*p* and *p*×*q* matrices of unknown regression coefficients, and *ε*_1_ and *ε*_2_ are random errors (which may also include regulation mechanisms not measured). Assume *n* iid subjects. Denote ***Y*** and ***X*** as the data matrices composed of the *Y*’s and *X*’s, respectively. For estimating the regression coefficient matrices, we consider a penalized approach, where the estimate of ***β***_***1***_ is defined as

6 $$ {}\hat{\boldsymbol{\beta_{1}}}=\underset{\boldsymbol{\beta}} {\text{argmin}}\left\{||\boldsymbol{Y}-\boldsymbol{X}\boldsymbol{\beta_{1}}||_{2}^{2}\,+\, \lambda\left((1\,-\,\alpha)||\boldsymbol{\beta_{1}}||^{2}_{2} \,+\, \alpha||\boldsymbol{\beta_{1}}||_{1}\right)\right\}.  $$

*λ*>0 and 0≤*α*≤1 are data-dependent tuning parameters, and ||·||_2(1)_ denotes the *ℓ*_2(1)_ norm. The estimate of ***β***_***2***_ can be defined in a similar manner. With the estimates, define $\boldsymbol {\hat {Y}}=\boldsymbol {X}\boldsymbol {\hat {\beta _{1}}}$ and $\boldsymbol {\hat {Z}}=\boldsymbol {Y}\boldsymbol {\hat {\beta _{2}}}$.

For $(\boldsymbol {X}, \boldsymbol {\hat {Y}}, \boldsymbol {\hat {Z}})$, the length *r*+*p*+*q* “mega” vector of omics measurements, define the (*r*+*p*+*q*)×(*r*+*p*+*q*) weight matrix

7 $$\begin{array}{@{}rcl@{}} \boldsymbol{\tilde{W}}= \left(\begin{array}{ccc} \boldsymbol{0} & \boldsymbol{W_{\hat{Z}:\hat{Y}}} & \boldsymbol{0}\\ \boldsymbol{W_{\hat{Z}:\hat{Y}}^{T}} & \boldsymbol{0} & \boldsymbol{W_{\hat{Y}:X}}\\ \boldsymbol{0} & \boldsymbol{W_{\hat{Y}:X}^{T}} & \boldsymbol{0} \end{array} \right), \end{array} $$

where ***0*** denotes a matrix with all components zero (note that in different places, it may have different dimensions), $\boldsymbol {W_{\hat {Z}:\hat {Y}}}$ and $\boldsymbol {W_{\hat {Y}:X}}$ are the matrices of similarity between $\boldsymbol {\hat {Z}}$ and $\boldsymbol {\hat {Y}}$, and between $\boldsymbol {\hat {Y}}$ and ***X*** respectively, and the superscript *T* denotes transpose.

For a partition of {1,…,*r*+*p*+*q*} (which leads to a clustering structure), using $\boldsymbol {\tilde {W}}$, we can compute the NCut measure NCut_*multi*_ in the same manner as in (1).

Rationale Linear regression has been adopted in multiple recent studies to describe the regulations between different types of omics measurements and shown to be effective [27, 28]. It has multiple desirable features including for example lucid interpretations, easy accommodation of multiple regulators (for each GE, protein), simple calculations, etc. We consider “unidirectional” effects, that is, from CNVs to GEs, and from GEs to proteins. We acknowledge that there can be “reversed” effects: for example, proteins may affect gene expression levels and methylation. However, such effects are usually much smaller, and accommodating them causes significant statistical challenges. For estimating the regulations (***β***’s), we adopt the elastic net (Enet) approach, which easily accommodates the sparsity of regulation relationships and correlations among regulators. Note that Enet is not essential here and can be replaced by other regularized estimation techniques. With the weight matrix properly constructed, we define NCut_*multi*_ in a way consistent with NCut_*single*_. Note that, although seemingly straightforward, NCut_*multi*_ (or similar quantities) has not been considered in the literature.

**MuNCut** With a fixed *K*, let *A*={*A*_1_,…,*A*_*K*_} denotes a disjoint partition of the CNVs, GEs, and proteins. Note that the cluster represented by *A*_*k*_ may contain multiple types of omics measurements. For *A*_*k*_ denote *A*_*k,C*_,*A*_*k,G*_, and *A*_*k,P*_ as its components that are CNVs, GEs, and proteins, respectively. For *A*, we define its MuNCut measure as

8 $$\begin{array}{@{}rcl@{}} \text{MuNCut}(A)=\text{NCut}_{multi}+\gamma \times \text{NCut}_{single}, \end{array} $$

where NCut_*multi*_ and NCut_*single*_ are as defined above, and *γ*≥0 is a tuning parameter. The optimal clustering is defined as the one that minimizes MuNCut(*A*).

Rationale The MuNCut objective function is the sum of two NCut ones. Its intuitive interpretation is similar to that of the standard NCut, that is, to minimize similarity across clusters and maximize similarity within clusters. Significantly advancing from NCut and other existing approaches, MuNCut considers both across- and within-cluster similarity for omics measurements of the same type as well as different types. We introduce *γ* to be more flexible and allow for different “degrees of emphasis” on within layers and across layers. For the example shown in Fig. 1, we present the MuNCut clustering result as well as that of the K-means. We observe that MuNCut accurately identifies the true clustering structure. In contrast, the K-means mostly separates different data types and fails to put different types of interconnected omics measurements in the same clusters. More definitive results are presented below in simulation.

**Remarks** When describing the regulations among omics measurements, we use a linear regression approach, which has been shown to be effective in the literature [20, 29]. Note that for the purpose of clustering, this regression (and Enet for its parameter estimation) is not essential. It can be replaced by other approaches, as long as a similarity measure can be generated. In addition, with the “scale-free” property of NCut, this similarity measure does not have to be consistent with that for within layers. For many types of omics measurements, the direction of regulation is clear. However, there are exceptions. For example, it is still not clear whether CNV and methylation regulate each other. Consider for example a dataset with methylation, CNV, GE, and protein measurements. We propose adopting an existing approach [30], stack the methylation and CNV measurements together, and create a vector of “mega regulators” (of GEs). The proposed approach can then be applied.

### Computation

**Computational algorithm** The proposed approach first involves computing the Enet estimates, which can be effectively realized using multiple existing techniques such as coordinate descent, which is adopted in the R package *glmnet* used in our numerical study.

For optimizing the MuNCut objective function, we adopt the simulated annealing (SA) technique [31]. At iteration *t*, denote $A^{(t)}=\left \{A_{1}^{(t)},\dots,A_{K}^{(t)}\right \}$ as the partition (clustering result) and MuNCut(*t*) as the value of the objective function. Further denote *B* as the maximum number of iterations. The value of *B* is not important, as long as it is large enough. Define the temperature function as *T*(*t*)=*L*log(*t*+1). In our numerical study, we set *L*=1000, which generates satisfactory result. In practice, to be prudent, other *L* values may also need to be examined. Discussions on tuning parameters with the SA technique are available in the literature and will not be reiterated. The proposed algorithm proceeds as follows.

**Step 1** Randomly initialize $A^{(0)}=\left \{A_{1}^{(0)},\dots,A_{K}^{(0)}\right \}$. In our numerical study, different initial values lead to similar results.

**Step 2** Set *t*=*t*+1. For *k*=1,…,*K*, compute *p*_*k*_ as the number of (*j,l*) pairs such that $j, l \in A_{k}^{(t-1)}$. Draw *k*(−) and *k*(+) from {1,…,*K*} with probabilities proportional and inversely proportional to *p*_*k*_.

**Step 3** Draw *i* randomly from $A_{k(-)}^{(t)}$. Set $A_{k(+)}^{(t)}=A_{k(+)}^{(t-1)}\cup \{i\}$ and $A_{k(-)}^{(t)}=A_{k(-)}^{(t-1)}\setminus \{i\}$. For *j*≠*k*(+),*k*(−), $A_{j}^{(t)}:=A_{j}^{(t-1)}$.

**Step 4** If MuNCut(*t*)≤MuNCut(*t*−1), then keep *A*^(*t*)^ as it is. If not, keep *A*^(*t*)^ as it is with probability $\exp \Bigg (\,-\,\frac {\text {MuNCut} (t)- \text {MuNCut} (t-1)}{T(t)} \!\Bigg)$, and otherwise *A*^(*t*)^=*A*^(*t*−1)^.

**Step 5** Repeat Steps 2-4 until *t*=*B*.

Extensive research on the SA technique is available in the literature [32, 33]. Briefly, in Step 2, the proposed probabilities prefer adding a new member to a small cluster and deleting a member from a large cluster. Thus, the “prior” is that clusters have similar sizes. Note that this strategy can be adjusted according to preference/prior information. Convergence of the SA algorithm to the global optimizer has been examined in the literature [34]. It is achieved in all of our numerical examples.

With the high efficiency of the coordinate descent and SA techniques, MuNCut is computationally very feasible. The two steps have computational complexity *O*(*nqpr*) and *O*(*Bqpr*), respectively. For a simulated dataset with *q*=*p*=*r*=200 and *n*=50, we consider 100 tuning parameter values in penalized estimation and *B*=10,000 in MuNCut. The proposed analysis takes about 30 s on a laptop with standard configurations.

**Tuning parameter selection** In the Enet penalization estimation, the tuning parameters are selected using cross validation, which is the default in *glmnet*. With the proposed MuNCut, the additional tuning parameters are *γ* (which balances single- and multi-layer NCut measures) and *K*, the number of clusters. For selecting these parameters, we adopt a cross validation-based approach [35], which has been developed in the context of biclustering and other studies and shown to be effective. Specifically, consider a (*γ*,*K*) dual. We randomly split data into a training set and a testing set. The MuNCut approach is applied to the training set. On the testing set, we predict GEs using CNVs in the same clusters and predict proteins using GEs in the same clusters. The overall prediction errors for GEs and proteins are then computed. Multiple splittings are conducted, and prediction errors are summed over splittings. The (*γ*,*K*) value that optimizes prediction is chosen as the optimal. In our simulation, this approach leads to satisfactory clustering results.

**Software development** To facilitate data analysis, we developed an R package *NCutYX* publicly available on CRAN at https://cran.r-project.org/web/packages/NCutYX/index.html. The proposed approach is implemented using the function *muncut*, which proceeds as follows: clust ← muncut(Z, Y, X, K =2, B =3000, L =1000, gamma =0.5, dist = “gaussian”, sigma = 1) In the above command, *Z* is the data matrix of proteins, *Y* is the data matrix of GEs, *X* is the data matrix of CNVs, *K* is the number of clusters, *B* is the number of SA iterations, *L* is the temperature coefficient, and gamma is the tuning parameter *γ*. The option dist selects the type of dissimilarity being used, which is the Gaussian kernel distance in this case with sigma specifying the bandwidth parameter. The resulting object *clust* is a list where the first entry (*clust[[1]]*) is a vector of SA sequence, and the second entry (*clust[[2]]*) includes the clustering results. The program can now accommodate three data layers. Researchers can easily modify the code to accommodate more layers.

## Results

### Data analysis

TCGA is a collaborative effort organized by NIH/NCI. For multiple cancer types, data have been collected on multiple types of genetic, epigenetic, genomic, and proteomic changes. With the high data quality and public availability, TCGA provides an ideal testbed for the proposed method. Here we analyze breast invasive carcinoma (BRCA) and cervical squamous cell carcinoma and endocervical adenocarcinoma (CESC) data. The processed level 3 data are downloaded using the R package gsdr. We refer to the TCGA website and published studies for more information on study design and data processing.

#### Evaluation measures

As the data generating mechanism is unknown, it is not possible to evaluate clustering accuracy. We conduct the following evaluation, which can provide some insights into the clustering results.

**Stability** We randomly select *n*/2 subjects without replacement [36] and analyze using the proposed as well as alternative methods. Repeat the process *N* times, and denote the adjacency matrix of the *k*th clustering as $\widehat {\boldsymbol {A}}^{(k)}$. Define the stability measure

9 $$ \boldsymbol{M}_{stability}=N^{-1}\sum_{1\leq k \leq N} \widehat{\boldsymbol{A}}^{(k)}.  $$

The (*i,j*)the element of this matrix describes the probability that the corresponding variables are clustered together. A stable approach has a large contrast: some elements have large values, and the others have very small values. The stability measure can be graphically presented using a heatmap, with warmer colors describing larger values (and colder colors describing smaller values). A heatmap with greater contrast is preferred.

**Concordance** When applying multiple methods to the same data, it is of interest to compare the similarity of analysis results. For two clustering methods *A* and *B*, denote the adjacency matrices as $\hat {\boldsymbol {A}}$ and $\hat {\boldsymbol {B}}$, respectively. Define the concordance of method *B* with respect to *A* as

10 $$ M(B|A)=\sum_{j,l}^{m}(\hat{\boldsymbol{A}}\odot \hat{\boldsymbol{B}})_{jl}/\sum_{j,l}^{m} (\hat{\boldsymbol{A}})_{jl},  $$

with a larger value suggesting higher similarity. Note that this concordance measure is not symmetric. That is, it is possible that *M*(*B*|*A*)≠*M*(*A*|*B*), and thus both values need to be calculated.

#### BRCA data

We analyze CNV, GE, and protein data. Data are collected and processed as follows. A quick examination of data suggests that there are much fewer protein measurements than for GEs and CNVs. Thus we first identify 873 subjects with 164 protein measurements. We then select the top 1000 GEs and CNVs with the strongest distance correlations [37] with the proteins. The considerations are that clustering is more sensible with correlated measurements, the numbers of GEs and CNVs relevant to proteins are not expected to be large, and (as observed in simulation) performance of the proposed method is better with a smaller number of variables. GEs and CNVs with missing measurements are removed from analysis. The three types of omics data are then merged together. The analyzed data contain 164 protein, 334 GE, and 514 CNV measurements on 873 subjects.

**Fig. 2:**
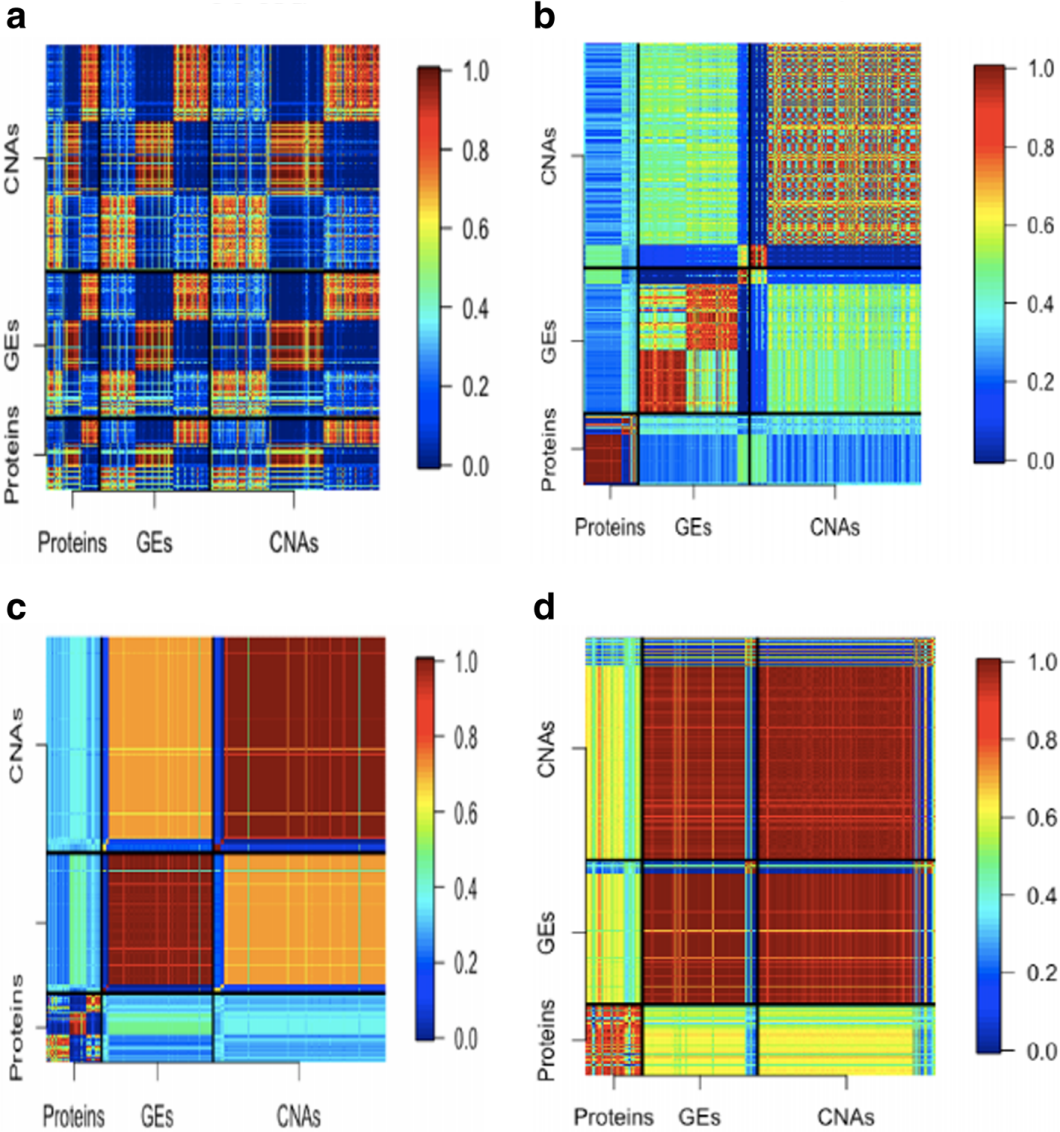
Analysis of BRCA data: stability of heatmaps. **a** MuNCut; **b** KM ^∗^; **c** SC ^∗^; **d** HC ^∗^. The (*i,j*)th entry is the probability that the *i*th and *j* elements belong to the same cluster. Higher/lower probabilities are presented using warmer/colder colors

With MuNCut and data-dependent tuning parameters, three clusters are generated. The detailed clustering results are presented in the Additional file 1. The numbers of (protein, GE, CNV) in the three clusters are (52, 112, 168), (55, 108, 168) and (57, 114, 179), respectively. Considering the inferior performance of KM, SC, HC, LC, and FGC observed in simulation, we here analyze data with KM*, SC*, and HC* and compare. The concordance results are presented in Table 1. More detailed clustering results using the alternatives are available from the authors. Table 1 suggests that the MuNCut results are moderately to strongly in concordance with those using the alternatives. Different methods generate different clustering results. The stability heatmaps of MuNCut and the three alternatives are shown in Fig. 2. Better stability results are observed for MuNCut. Specifically, very warm colors are observed within clusters, and very cold colors are observed across clusters. This is not observed with the alternatives. In addition, a closer examination suggests that the alternatives often generate one big cluster along with very small clusters, which can be less interpretable and hence not desired.

**Table 1:** Data analysis: concordance between the analysis results using different methods. In each cell, *M*(B |A), where B and A are the clustering methods in the column and row, respectively

BRCA	MuNCut	KM*	SC*	HC*
MuNCut	100%	59.4%	72.7%	80.1%
KM*	44.7%	100%	74%	82.3%
SC*	34.5%	46.7%	100%	90.1%
HC*	36.3%	49.6%	85.9%	100%
CESC	MuNCut	KM*	SC*	HC*
MuNCut	100%	48.3%	44.6%	52.5%
KM*	37.8%	100%	51.3%	64.7%
SC*	38.7%	56.9%	100%	61.5%
HC*	35.6%	56.2%	48.1%	100%

#### CESC data

**Fig. 3:**
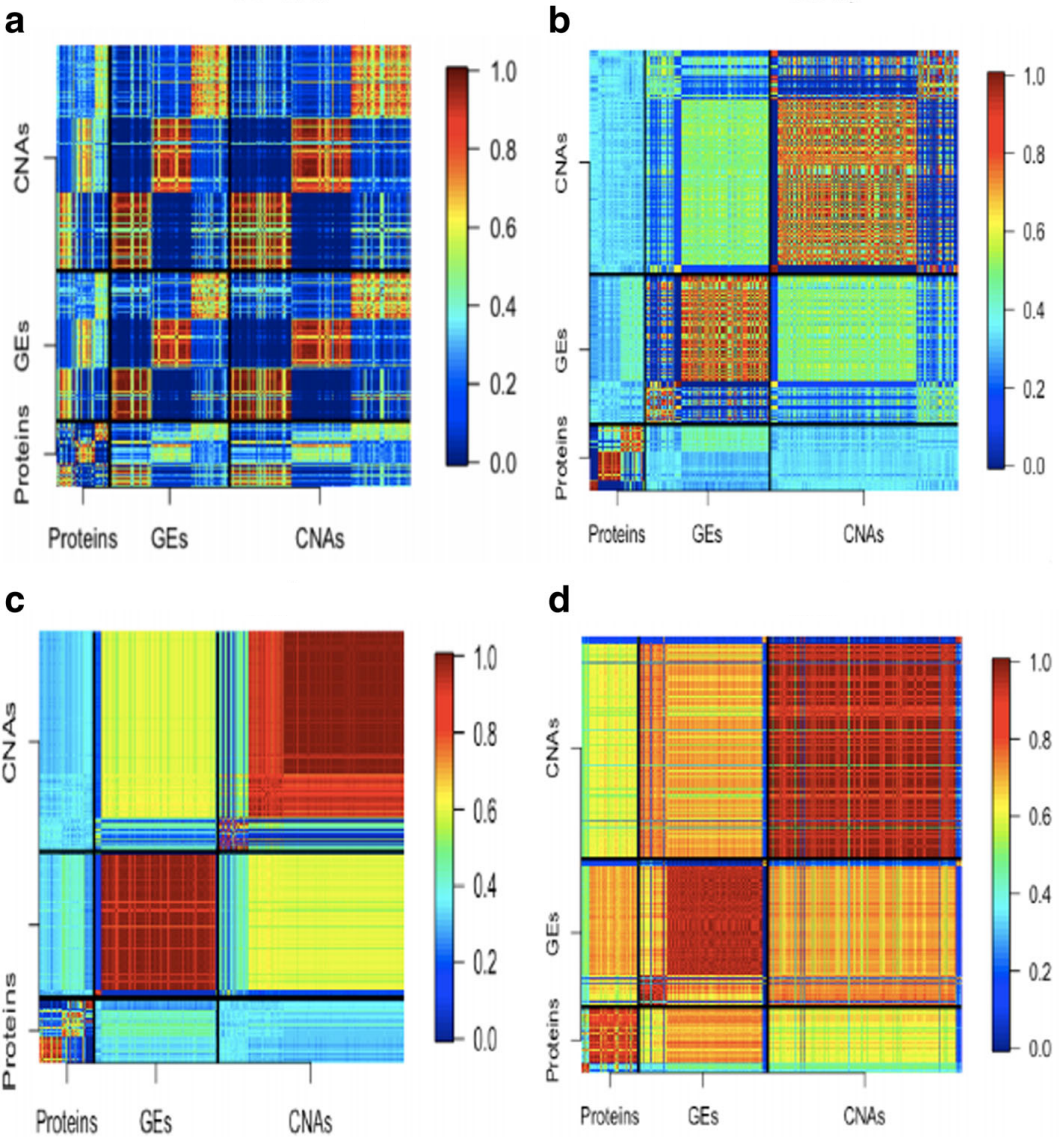
Analysis of CESC data: stability of heatmaps. **a** MuNCut; **b** KM ^∗^; **c** SC ^∗^; **d** HC ^∗^. The (*i,j*)th entry is the probability that the *i*th and *j* elements belong to the same cluster. Higher/lower probabilities are presented using warmer/colder colors

We first conduct the same data collection and processing as previously described. The analyzed data contain 144 protein, 325 GE, and 488 CNV measurements on 164 subjects. When employing the proposed method, three clusters are generated. The detailed clustering results are presented in the Additional file 1. The numbers of (protein, GE, CNV) in the three clusters are (45, 100,160), (43, 104, 152) and (56, 121, 176), respectively. The concordance analysis in Table 1 again suggests that MuNCut generates results different from using the alternatives, and different methods have moderate concordance. More detailed clustering results using the alternatives are available from the authors. The stability heatmaps are presented in Fig. 3. For MuNCut, we again observe an obvious contrast between warm/cold color regions, which suggests satisfactory stability. More closely examining the numerical values suggests that the stability is lower than for the BRCA data, which is reasonable with the smaller sample size. For the alternatives, observations similar to those for the BRCA data are made.

### Simulation

We conduct simulation to gauge performance of the proposed approach and compare against multiple relevant alternatives.

**Alternative methods** In the literature, methods tailored to the present data settings are lacking. We consider the following alternatives because of their popularity and relevance. It is specially noted that methods for clustering subjects (with multilayer omics measurements) are not applicable for clustering omics measurements. We first consider clustering using the standard K-means (KM), spectral clustering (SC), and hierarchical clustering (HC). These methods are directly applied to the pooled data, i.e, (*Z,Y,X*). Note that this approach does not take into account the differences among multiple types of omics measurements. To tackle this problem, we also consider KM*, SC*, and HC*, the matching version of these three methods. Consider for example KM*. The KM clustering is first conducted with the three types of measurements separately. Here we reinforce that the numbers of clusters for all three data types are the same. We then match clusters across layers. Specifically, we experiment with all combinations of CNV, GE, and protein clusters, and select the one with the strongest associations between GE and CNV clusters and between protein and GE clusters. This approach is built on the popular clustering techniques and conducts “post-clustering connections” across layers. A potential advantage of this approach is that if clusters from different layers “confirm” each other, then the results can be more robust and trustworthy. In addition, this approach can potentially avoid the “overfitting” problem with simultaneously clustering multiple layers (as for example the connections among GEs can be attributable to connections among CNVs and GE-CNV regulations). With the growing popularity of network analysis, we also consider two network community detection methods, namely the Louvain (LC) [38] and Fast Greedy Clustering (FGC) [39] methods, which have demonstrated competitive performance in the literature. For the fairness of comparison, we use the same network and similarity matrix input as for the MuNCut. As such, this comparison can directly reveal the advantage of the proposed clustering.

**Evaluation of clustering accuracy** For a specific clustering result {*A*_1_,…,*A*_*K*_}, an adjacency matrix ***A***=(*a*_*jl*_)_*m*×*m*_ (*m*=*q*+*p*+*r*) can be constructed, where the element *a*_*jl*_=1 if the omics measurements corresponding to *j* and *l* belong to the same cluster, and *a*_*jl*_=0 if otherwise. Let ***A***_*T*_ and $\hat {\boldsymbol {A}}$ be the adjacency matrices of the true and estimated clusters, respectively. Then the accuracy measure is defined as the diversity between ***A***_*T*_ and $\hat {\boldsymbol {A}}$,

11 $$ M_{accuracy}=1-\sum_{j,l}^{m}(\boldsymbol{A}_{T} \odot \hat{\boldsymbol{A}})_{jl}/m^{2},  $$

where ⊙ is the component-wise product. A smaller value indicates more accurate clustering.

**Scenario I** Set *p*=*q*=*r*. We have also examined settings with unequal sizes and observed similar performance (results omitted). There are four clusters, and the numbers of CNVs (GEs, proteins) in the four clusters are *p*/5,2*p*/5,*p*/5,*p*/5, respectively. The clustering structure is determined by the distribution of CNVs and the two regression models. Specifically, *X* is generated from a multivariate normal distribution with marginal means zero, marginal variances one, and variance-covariance matrix ***Σ***. ***Σ*** has a block diagonal structure. The first three blocks, which correspond to the first three clusters, have off diagonal elements all equal to *ρ*. Two different *ρ* values are considered (0.2 and 0.4), representing weak and moderate correlations. The fourth block, which corresponds to the last cluster, is identity. Under this correlation structure, CNVs in different clusters are independent of each other. For the first three clusters, CNVs within the same cluster are correlated. For the fourth cluster, CNVs within the same cluster are independent. ***β***_***1***_ and ***β***_***2***_ also have the same block diagonal structure as ***Σ***. That is, GEs only depend on CNVs in the same cluster; and proteins only depend on GEs in the same cluster. For the first three clusters, 20% of randomly selected elements are nonzero and have values satisfying *Unif*(*h*/2,*h*). Two different *h* values are considered, representing weak and moderate regulations. The remaining 80% of the elements are zero, corresponding to sparse regulations. Note that, this data generating mechanism allows for both cis- and trans-acting effects. For the fourth cluster, the corresponding blocks in ***β***_***1***_ and ***β***_***2***_ have all zero elements. For the first three clusters, measurements in the same cluster are interconnected with each other and not connected with measurements in other clusters. The fourth cluster is a “noisy” cluster, which reflects the “biological reality” that there are some “isolated” omics measurements, and some GEs and proteins have regulations too weak to be detected. In the two regression models, the random errors are generated from *N*(0,1).

For each setting, 200 replicates are generated. The results are summarized in Table 2. Across all simulation settings, the proposed method outperforms the eight alternatives. For example in the first row, with *n*=200,*q*=400,*h*=0.15, and *ρ*=0.20, MuNCut has *M*_*accuracy*_ value 0.023, compared to 0.411 (KM), 0.47 (SC), 0.565 (HC), 0.13 (KM*), 0.126 (SC*), 0.159 (HC*), 0.155 (LC), and 0.157 (FGC). The classic KM, SC, and HC consistently perform poorly. A closer examination of the analysis results suggests that their clusters tend to include just a single type of omics data. That is, they fail to cluster interconnected CNVs and GEs and proteins together. The matching versions KM*, SC* and HC* can solve this problem to a certain extent. However, they are still inferior to the proposed method. The network-based methods LC and FGC have stable performance across settings, however, inferior to the proposed method. When the dimensionality is high (*q*=*p*=*r*=1200), performance of the proposed method deteriorates. This is reasonable, as the proposed method needs to estimate the regulations where the number of parameters grows quadratically. When the data dimensionality is ultrahigh (for example in a whole-genome study), it is usually possible to select a smaller number of “interesting” genes for analysis. Another option is to use biological (for example pathway) or statistical information, separate measurements into smaller functional sets, and conduct clustering analysis with each set separately.

**Table 2:** Simulation results for Scenario I

Parameters				*M*_*accuracy*_								
*n*	*q*	*h*	*ρ*	MuNCut	KM	SC	HC	KM*	SC*	HC*	LC	FGC
200	400	0.15	0.20	0.023	0.411	0.47	0.565	0.13	0.126	0.159	0.155	0.157
200	400	0.15	0.40	0.016	0.364	0.468	0.585	0.134	0.115	0.17	0.160	0.159
200	400	0.25	0.20	0.054	0.368	0.474	0.587	0.131	0.123	0.157	0.155	0.152
200	400	0.25	0.40	0.068	0.363	0.477	0.586	0.133	0.117	0.193	0.151	0.149
400	400	0.15	0.20	0.022	0.373	0.460	0.588	0.129	0.124	0.165	0.160	0.159
400	400	0.15	0.40	0.014	0.364	0.468	0.585	0.129	0.123	0.174	0.160	0.160
400	400	0.25	0.20	0.048	0.367	0.462	0.586	0.127	0.115	0.175	0.153	0.151
400	400	0.25	0.40	0.063	0.361	0.464	0.584	0.12	0.11	0.176	0.148	0.147
200	800	0.15	0.20	0.095	0.322	0.44	0.576	0.122	0.124	0.152	0.150	0.149
200	800	0.15	0.40	0.103	0.319	0.432	0.575	0.127	0.129	0.173	0.146	0.145
200	800	0.25	0.20	0.111	0.33	0.366	0.582	0.126	0.123	0.153	0.141	0.162
200	800	0.25	0.40	0.128	0.315	0.433	0.577	0.129	0.134	0.17	0.134	0.138
400	800	0.15	0.20	0.092	0.318	0.423	0.577	0.134	0.114	0.168	0.148	0.148
400	800	0.15	0.40	0.102	0.324	0.428	0.579	0.111	0.107	0.149	0.143	0.143
400	800	0.25	0.20	0.109	0.319	0.431	0.579	0.119	0.115	0.162	0.138	0.154
400	800	0.25	0.40	0.123	0.324	0.427	0.58	0.135	0.139	0.174	0.188	0.133
200	1200	0.15	0.20	0.11	0.312	0.384	0.578	0.139	0.139	0.157	0.145	0.156
200	1200	0.15	0.40	0.104	0.304	0.395	0.577	0.135	0.14	0.17	0.138	0.144
200	1200	0.25	0.20	0.124	0.308	0.4	0.576	0.132	0.131	0.153	0.212	0.162
200	1200	0.25	0.40	0.131	0.309	0.395	0.582	0.133	0.136	0.168	0.207	0.212
400	1200	0.15	0.20	0.112	0.316	0.388	0.58	0.122	0.124	0.154	0.141	0.154
400	1200	0.15	0.40	0.122	0.314	0.396	0.58	0.123	0.123	0.161	0.160	0.126
400	1200	0.25	0.20	0.127	0.315	0.403	0.573	0.13	0.132	0.162	0.186	0.197
400	1200	0.25	0.40	0.127	0.309	0.40	0.579	0.135	0.137	0.173	0.157	0.231

*n is the sample size; q is the number omics measurements in each layer;*

*h measures the strength of regulation across layers;* *ρ* *is the correlation coefficient among CNVs*

**Scenario II** In the above simulation, CNVs, GEs, and proteins not in the same cluster are independent, which can be too simplified. Here we consider a more realistic scenario. The settings are mostly identical to those in the previous simulation. The key difference is in the variance-covariance matrix ***Σ***. Specifically, the first three clusters have off diagonal elements equal to 2*ρ*, and all other off diagonal elements of ***Σ*** are equal to *ρ*. That is, for the first three clusters, CNVs, GEs, and proteins in the same cluster are more strongly interconnected. However, those in the fourth cluster and those in different clusters are still correlated. This setting has many more correlations and is more challenging than the previous one.

The results are summarized in Table 3. The patterns are similar to those observed in Table 2. Under most settings, the proposed method outperforms the eight alternatives. Under a small number of settings, it is only slightly inferior to SC*, with very small differences.

**Table 3:** Simulation results for Scenario II

Parameters				*M*_*accuracy*_								
*n*	*q*	*h*	*ρ*	MuNCut	KM	SC	HC	KM*	SC*	HC*	LC	FGC
200	400	0.15	0.20	0.026	0.365	0.462	0.582	0.13	0.122	0.188	0.139	0.155
200	400	0.15	0.40	0.025	0.411	0.476	0.564	0.133	0.119	0.202	0.158	0.157
200	400	0.25	0.20	0.095	0.409	0.475	0.566	0.131	0.122	0.19	0.163	0.163
200	400	0.25	0.40	0.118	0.409	0.473	0.563	0.124	0.12	0.202	0.157	0.155
400	400	0.15	0.20	0.024	0.412	0.469	0.564	0.13	0.125	0.204	0.155	0.152
400	400	0.15	0.40	0.024	0.413	0.475	0.567	0.129	0.123	0.197	0.155	0.153
400	400	0.25	0.20	0.096	0.413	0.469	0.564	0.128	0.113	0.20	0.162	0.159
400	400	0.25	0.40	0.111	0.411	0.479	0.565	0.125	0.134	0.203	0.153	0.151
200	800	0.15	0.20	0.113	0.399	0.436	0.561	0.129	0.118	0.179	0.152	0.174
200	800	0.15	0.40	0.132	0.397	0.443	0.560	0.138	0.138	0.194	0.144	0.143
200	800	0.25	0.20	0.132	0.405	0.432	0.562	0.127	0.12	0.18	0.181	0.151
200	800	0.25	0.40	0.142	0.397	0.442	0.56	0.138	0.137	0.197	0.208	0.164
400	800	0.15	0.20	0.106	0.402	0.443	0.560	0.129	0.129	0.184	0.148	0.149
400	800	0.15	0.40	0.134	0.394	0.452	0.559	0.14	0.137	0.198	0.141	0.140
400	800	0.25	0.20	0.13	0.391	0.431	0.546	0.125	0.122	0.189	0.180	0.149
400	800	0.25	0.40	0.141	0.396	0.429	0.561	0.143	0.142	0.196	0.165	0.213
200	1200	0.15	0.20	0.127	0.383	0.412	0.554	0.137	0.131	0.161	0.145	0.176
200	1200	0.15	0.40	0.143	0.404	0.441	0.558	0.14	0.138	0.186	0.218	0.149
200	1200	0.25	0.20	0.137	0.393	0.417	0.558	0.142	0.14	0.178	0.224	0.219
200	1200	0.25	0.40	0.148	0.393	0.434	0.56	0.141	0.14	0.188	0.163	0.238
400	1200	0.15	0.20	0.126	0.398	0.426	0.559	0.14	0.142	0.183	0.194	0.147
400	1200	0.15	0.40	0.14	0.396	0.427	0.559	0.142	0.141	0.184	0.192	0.221
400	1200	0.25	0.20	0.126	0.401	0.428	0.560	0.139	0.141	0.181	0.165	0.220
400	1200	0.25	0.40	0.142	0.397	0.420	0.559	0.143	0.147	0.187	0.165	0.242

*n is the sample size; q is the number omics measurements in each layer;*

*h measures the strength of regulation across layers;* *ρ* *is the correlation coefficient among CNVs*

**Scenario III** Under this scenario, the true data generating models are

12 $$\begin{array}{@{}rcl@{}} Y=X\boldsymbol{\beta_{1}}+U_{1}\boldsymbol{\gamma_{1}}+\epsilon_{1}, ~~ Z=Y\boldsymbol{\beta_{2}}+U_{2}\boldsymbol{\gamma_{2}}+\epsilon_{2}. \end{array} $$

Here *U*_1_ and *U*_2_ are length *s*_1_ and *s*_2_ vectors and describe regulating mechanisms that also affect *Y* and *Z* but are not measured, reflecting the fact that, in some studies, data collection can be “incomplete”, and not all relevant regulators are measured. ***γ***_***1***_ and ***γ***_***2***_ are matrices of regression coefficients. In simulation, we set *s*_1_=*s*_2_=*p*, generate *U*_1_ and *U*_2_ in the same way as *X*, and generate ***γ***_***1***_ and ***γ***_***2***_ in the same way as ***β***_***1***_ and ***β***_***2***_. Note that under this data generating mechanism, models in (5) are mis-specified.

Results for this specially challenging scenario are summarized in Table 4. With the mis-specified models, performance of MuNCut is not as competitive as in the previous simulations. However, for two-thirds of the simulation settings, it still outperforms the alternatives, sometimes by a large margin. For the remaining settings, its performance is very close to the best alternative.

**Table 4:** Simulation results for Scenario III

Parameters				*M*_*accuracy*_								
*n*	*q*	*h*	*ρ*	MuNCut	KM	SC	HC	KM*	SC*	HC*	LC	FGC
200	400	0.15	0.20	0.064	0.359	0.459	0.583	0.124	0.125	0.186	0.172	0.200
200	400	0.15	0.40	0.108	0.354	0.464	0.582	0.124	0.126	0.194	0.171	0.171
200	400	0.25	0.20	0.126	0.360	0.462	0.584	0.127	0.128	0.186	0.192	0.224
200	400	0.25	0.40	0.141	0.355	0.468	0.578	0.131	0.129	0.198	0.147	0.144
400	400	0.15	0.20	0.06	0.356	0.457	0.583	0.121	0.123	0.185	0.171	0.169
400	400	0.15	0.40	0.097	0.354	0.46	0.587	0.12	0.124	0.193	0.164	0.162
400	400	0.25	0.20	0.121	0.358	0.456	0.585	0.122	0.123	0.185	0.174	0.152
400	400	0.25	0.40	0.138	0.357	0.462	0.586	0.124	0.124	0.191	0.134	0.136
200	800	0.15	0.20	0.122	0.314	0.434	0.578	0.13	0.132	0.189	0.175	0.172
200	800	0.15	0.40	0.134	0.315	0.431	0.579	0.139	0.134	0.19	0.212	0.172
200	800	0.25	0.20	0.142	0.32	0.402	0.567	0.128	0.128	0.19	0.202	0.195
200	800	0.25	0.40	0.144	0.318	0.414	0.58	0.146	0.144	0.196	0.166	0.206
400	800	0.15	0.20	0.121	0.321	0.421	0.578	0.129	0.129	0.174	0.191	0.153
400	800	0.15	0.40	0.144	0.321	0.427	0.577	0.146	0.144	0.193	0.165	0.216
400	800	0.25	0.20	0.141	0.315	0.424	0.578	0.127	0.128	0.173	0.152	0.197
400	800	0.25	0.40	0.143	0312	0.439	0.579	0.131	0.134	0.188	0.168	0.228
200	1200	0.15	0.20	0.138	0.307	0.391	0.578	0.139	0.139	0.168	0.207	0.212
200	1200	0.15	0.40	0.146	0.314	0.389	0.575	0.148	0.147	0.19	0.160	0.235
200	1200	0.25	0.20	0.136	0.30	0.374	0.575	0.136	0.133	0.169	0.187	0.225
200	1200	0.25	0.40	0.144	0.308	0.405	0.572	0.146	0.145	0.189	0.169	0.232
400	1200	0.15	0.20	0.136	0.316	0.406	0.573	0.138	0.139	0.163	0.159	0.223
400	1200	0.15	0.40	0.144	0.30	0.389	0.571	0.146	0.145	0.189	0.165	0.239
400	1200	0.25	0.20	0.141	0.316	0.376	0.577	0.135	0.139	0.183	0.171	0.228
400	1200	0.25	0.40	0.141	0.308	0.391	0.575	0.139	0.14	0.186	0.146	0.219

*n is the sample size; q is the number omics measurements in each layer;*

*h measures the strength of regulation across layers;* *ρ* *is the correlation coefficient among CNVs*

## Discussion

Clustering analysis results can be used in multiple ways. For example, they can suggest the functional connections among measurements. As can be partly seen in Fig. 1, by taking into account the interconnections across layers, the MuNCut results look like *channels*: from CNVs to their regulated GEs, and from GEs to their encoded proteins. The MuNCut results can be biologically more informative. In the literature, clustering results have also been used to assist dimension reduction in model building. Recent studies have also conducted model building using multilayer omics data. It can be of interest to explore using the MuNCut results in such analysis. The proposed method can be potentially extended. Presently, the regulation relationships are built purely statistically. For the regulation of GEs and coding of proteins, there exists extensive biological information accumulated from functional experiments. It can be of interest to accommodate some of that information. We do note that such information is still partial and cannot completely replace the proposed statistical analysis. In our description and data analysis, CNV, GE, and protein are used. The proposed method can directly accommodate more/other types of omics data. It can be of interest to conduct more extensive data analysis with additional omics measurements. In data analysis, results different from using the alternatives are obtained. However, additional experiments or studies may be needed to fully validate our findings. The stability evaluation results and superior performance observed in simulation may to a certain extent suggest the credibility of our analysis.

## Conclusion

With omics data, clustering analysis has played an important role. Significantly advancing from some of the existing studies, we have developed a novel clustering method tailored to multilayer omics data. For quite a few complex diseases, recent multilayer omics studies have provided important insights not shared by the single-layer studies. This study has filled the knowledge gap by being among the first to develop tailored clustering methods that can informatively accommodate connections not only within layers but also across layers. The proposed method has an intuitive formulation and can be effectively realized using an SA algorithm. Across a wide spectrum of simulation settings, it significantly outperforms multiple relevant competitors. In the analysis of TCGA datasets, it leads to clustering results different from using the alternatives and with satisfactory stability.

## Additional files

Additional file 1 The file SupplementaryFile.xlsx contains detailed MuNCut clustering results for BRCA and CESC data. (XLSX 17 kb)

## Abbreviations

BRCA: Breast invasive carcinoma
CESC: Cervical squamous cell carcinoma and endocervical adenocarcinoma
CNV: Copy number variation
GE: Gene expression
HC: Hierarchical clustering
HC ^∗^: Matching hierarchical clustering
KM: K-means
KM ^∗^: Matching K-means
MuNCut: Multilayer normalized cut
NCut: Normalized cut
SA: Simulated annealing
SC: Spectral clustering
SC ^∗^: Matching spectral clustering
TCGA: The cancer genome atlas
